# The disparity mutagenesis model predicts rescue of living things from catastrophic errors

**DOI:** 10.3389/fgene.2014.00421

**Published:** 2014-12-04

**Authors:** Mitsuru Furusawa

**Affiliations:** Neo-Morgan Laboratory IncorporatedKawasaki, Japan

**Keywords:** disparity mutagenesis, DNA replication, lagging, leading, error-threshold, human evolution

## Abstract

In animals including humans, mutation rates per generation exceed a perceived threshold, and excess mutations increase genetic load. Despite this, animals have survived without extinction. This is a perplexing problem for animal and human genetics, arising at the end of the last century, and to date still does not have a fully satisfactory explanation. Shortly after we proposed the disparity theory of evolution in 1992, the disparity mutagenesis model was proposed, which forms the basis for an explanation for an acceleration of evolution and species survival. This model predicts a significant increase of the mutation threshold values if the fidelity difference in replication between the lagging and leading strands is high enough. When applied to biological evolution, the model predicts that living things, including humans, might overcome the lethal effect of accumulated deleterious mutations and be able to survive. Artificially derived mutator strains of microorganisms, in which an enhanced lagging-strand-biased mutagenesis was introduced, showed unexpectedly high adaptability to severe environments. The implications of the striking behaviors shown by these disparity mutators will be discussed in relation to how living things with high mutation rates can avoid the self-defeating risk of excess mutations.

## Introduction

The number of mutations per generation per diploid was calculated for several species, and found to range widely: nematode (0.32), drosophila (2.8), mouse (60), and human (128) (Drake et al., 1998). At this time, the average number of mutations introduced per one generation in human was estimated by indirect methods. In one method, the number of cell-cycles from a zygote to the next generation's zygote and the average mutation rate per one cell-cycle were used. With a second method, the number of SNPs (single nucleotide polymorphisms) between chimpanzee and human, their generation times and the phylogenetic relation between both species were used. These two indirect approaches gave nearly equal values; 135 ± 25 mutations per generation per diploid (Kondrashov, 1995; Crow, 1997; Drake et al., 1998; Eyre-Walker and Keightley, 1999; Nuchman and Crowell, 2000). Subsequently, using direct sequencing methods, spontaneous mutations between parents and their children were detected on a genome-wide scale, resulting in the smaller numbers, closer to 56~77 mutations per generation per diploid (Roach et al., 2010; Conrad et al., 2011; Campbell et al., 2012; Kong et al., 2012). In one study a higher number of 100 mutations per generation per diploid was reported (Xue et al., 2009). At the present time, it is believed that the number of mutations per generation per diploid in human is ~70 (Keightley, 2012). This assumption can also be demonstrated by the following calculation. With a human mutation rate considered to be about 10^9^ per site per year, genome size—about 3 × 10^9^ bp and average generation time—20~25 years, the human mutation number per generation can be estimated with a value between 60 and 75. Therefore, the mentioned above number of mutations per generation per diploid about 70 lies within this range.

From the viewpoint of phylogenetics, this two-fold difference in mutation rates becomes important, because it critically influences the determination of the branching period of both chimpanzee and human species. In contrast, when we consider the relationship between mutation rates and the extinction of populations, this two-fold difference can be ignored, since the values of mutations per generation obtained from the direct and indirect methods are far above the so-called “threshold.” The meaning of the term threshold, which is Eigen's threshold, will be explained below.

In this article, we would like to consider the distinction of population to focus on error (mutation)-threshold. Quasi-species is a conceptual species in chemistry, in which DNA or RNA population is produced by means of mutation and selection (Eigen et al., 1989). The error threshold means the maximum allowable value of mutation rate, when a quasi-species climbs up a fitness-landscape. In other words, when the mutation rate/base is less than the reciprocal number of the length of the genomic base sequence (i.e., the mutation number/replication/genome <1), the quasi-species can evolve. When mutation rates overstep this value (Eigen's error-threshold), a quasi-species model shows that the population becomes extinct due to the self-destruction of genetic information (Eigen et al., 1989; Nowak, 2006). The term self-destruction of genetic information means that genetic information itself becomes meaningless merely due to excess accumulation of mutations, consequently, these individuals cannot survive. Humans have the highest mutation rate/generation as far as we know. How can we survive with these extremely high mutation rates? According to Drake et al., the number of deleterious mutations is 1.8 in mice and 3.2 in humans (Drake et al., 1998). Here, however, we use the number of deleterious mutations at 2.2 per generation (Keightley, 2012). Judging by the knowledge of traditional population genetics, humans should already have too heavy a genetic load and be extinct. This is one of the biggest questions in human population genetics today and is frequently discussed (Crow, 2000; Charlesworth, 2012, 2013; Keightley, 2012; Lesecque et al., 2012; Scally and Durbin, 2012).

When considering this, is the premise correct that living things are inevitably exposed to the danger of extinction when mutation rates exceed the threshold? When tracing the cause of a mutational incident, it is generally believed that spontaneous mutations are evenly introduced into two daughter DNAs independently from the lagging and the leading strand. This assumption, which appears to be relatively unchallenged, should be called into question. Evenly distributed spontaneous mutations do not necessarily mean that the mutations are introduced evenly in the process of DNA replication. For instance, if biased-mutagenesis occurs in either the lagging or leading strand in every replication, evidence of biased-mutagenesis can be hard to discover by analysis of SNPs between two individuals. This is because the basic biased-partitioning of spontaneous mutations in replication will become equalized after repeated semiconservative DNA replications (Iwaki et al., 1996).

Base mismatching errors accompanying DNA replication mainly give rise to SNPs and are a root cause of evolution. Therefore, we focused on the molecular mechanism of DNA replication, in particular to the asymmetrical structure of the DNA-replication machinery. Our disparity mutagenesis model, includes the proposition that lagging strand biased mutagenesis occurs in every replicore in every cell division. In such an unbalanced situation, we demonstrated that living things can overcome the fatal effects of deleterious mutations by increasing the threshold of mutation (Furusawa and Doi, 1992, 1998).

The present article consists of the following sections. (1) Reasons for why living things can survive irrespective of high mutation rates; (2) How the disparity mutagenesis increases the threshold of mutation rates; (3) Acceleration of evolution using digital organisms with disparity mutagenesis; (4) Acceleration of evolution using disparity-mutators of living microorganisms; and (5) Discussions and conclusive remarks.

## Previous arguments for why humans can survive irrespective of high mutation rates

As mentioned above, depending on the methodologies used the number of mutations per generation in human varies in some degree, from 56 to 175 (Kondrashov, 1995; Crow, 1997; Drake et al., 1998; Eyre-Walker and Keightley, 1999; Nuchman and Crowell, 2000; Roach et al., 2010; Conrad et al., 2011; Campbell et al., 2012; Kong et al., 2012). Yet, irrespective of the excess accumulation of mutations per generation, humans continue to exist. This simple but significant discrepancy has been addressed differently from the viewpoint of natural selection in population genetics.

### Effect of population size

In human, selection against deleterious mutations is not as strong as thought; 30–42% of amino acid changing mutations are weakly deleterious mutations, 27–29% are neutral or nearly neutral and the remainders are highly deleterious. This is because humans' selection coefficient (s) is small mainly due to the small population size (Ne = 10,000 at 800,000 years ago) (Boyko et al., 2008).

### Truncation selection

Truncation selection allows only individuals with highest trait values to produce (Barton et al., 2007). The reduction of fitness is expressed by genetic load L = 1−e^−U^, where U is the number of deleterious mutations that are added in each generation (Kimura and Maruyama, 1966). When *U* is 0, *L* = 0. No reduction of fitness occurs. When U is enough high, L is close to 1. This indicates that the extinction of population occurs. Deleterious mutations, which are added in every generation, might significantly decrease relative fitness by synergistic epistasis, resulting in the chance that a majority of individuals might accumulate an excess number of deleterious mutations. In order to keep the population size, these individuals must be cut off by truncation selection (Crow and Kimura, 1979; Barton et al., 2007). As a result, deleterious mutations are effectively eliminated from the population.

Moreover, an efficacious increase in this genetic cleanup would be expected owing to positive epistasis among deleterious mutations (Nuchman and Crowell, 2000). Individuals who slipped through the truncation must immediately shoulder a burden of deleterious mutations. Notably, a positive epistasis among deleterious mutations seems to be rare (Kouyos et al., 2007). Thus, the average fitness of this population may decrease with time, and to keep the population size under such a severe situation, surviving individuals must increase their reproduction rate toward the corresponding death rate. Advantageous mutations may be minimally helpful for increasing the reproductive rate of surviving individuals because the incidence of advantageous mutations is though to be extremely low (Boyko et al., 2008). In conclusion, it is likely that truncation selection is still not sufficient to explain how humans are rescued from extinction.

On the other hand, Crow emphasized the contributions of sex (Crow, 2000). Sex shuffles genes within a population, resulting in an increased chance of producing individuals with accumulated deleterious mutations, but these could be effectively eliminated from the population via their death or loss of breeding potential. Yet, how can the population size be sustained in the connection of sex and natural selection? For that, a concept named “quasi-truncation selection” was applied, in which mildly deleterious mutations were considered (Crow and Kimura, 1979; Crow, 2000). Certainly, genetic shuffling via sex might have merit for decreasing the harmful effects of mutations, but will this prevent decreasing of fitness in an actual human population? The human mutation rate was not necessarily always the same as it is today, and future human mutation rates might increase ten or more times (Lesecque et al., 2012). These problems will be discussed later.

### Relative selection

When *U* = 2.2 in humans, *L* = 0.89. Humans seem to be intolerant of this deleterious mutation when selection is strong. However, if natural selection pressure act on relative fitness differences between individuals, the population will continue to exist through competitions among individuals. Unlike a direct selection pressure from environment, the selection pressure from different individuals which is caused by the fitness difference between individuals would be weak. The results of simulations based on this idea indicated that even when 10~100 deleterious mutations per generation per diploid are introduced, humans do not become extinct (Lesecque et al., 2012).

Considering functionally important sites, where natural selection may be weak, we can estimate U and L to be around 0.35 and 0.3, respectively. Thus, humans might overcome this range of genetic load (Charlesworth, 2012; Lesecque et al., 2012).

### Stabilizing selection

Recent studies on human spontaneous mutations show that the rates of both advantageous and deleterious mutations are higher than expected (Halligan and Keightly, 2006; Eory et al., 2010; Zeng and Charlesworth, 2010; Ward and Kellis, 2012). These evidences cannot be explained by purifying selection because purifying selection refers to directional selection against deleterious mutations. Thus, in humans, it is presumed that only very weak purifying selection may act on non-synonymous and 7- to 8-digit number of silent-site mutations. Under this assumption, weak purifying selection will allow to maintain the damaged genes which were formerly advantageous before the mutation. As a result, the variation of advantageous genes contributing to the increase of fitness for quantitative trait (genetic variance in fitness) is reduced significantly, and at the same time, genetic load comes close to 1 by accumulating deleterious mutations. Thus, purifying selection may not contribute to preservation of the species (Charlesworth and Charlesworth, 2010; Charlesworth, 2013).

On the other hand, when weak stabilizing selections act on an excessively large number of sites scattered throughout the genome, the species merely takes a mild genetic load, resulting in evoking a mild genetic variance in fitness. For instance, when 10% of non-coding genomic regions (10^8^ sites) receive stabilizing selection, the genetic load is estimated to be 0.05 (population size: Ne = 10,000 and the rate of heterozygote: p = 0.001). Therefore, the human population can tolerate to the genetic load (Kong et al., 2012). In this regard, when Ne is adequately large, L becomes too large to ignore (Charlesworth, 2013). At any rate, it seems likely that the direct application of traditional models of population genetics to human populations would be stretching things a bit. The main reason would be an unpredictable effect of genetic drift on evolution due to the small population size of humans.

## How the disparity-mutagenesis model increases the threshold of mutation rates

The disparity mutagenesis model is deduced from the principles of the “Disparity Theory of Evolution” (Furusawa and Doi, 1992, 1998; Furusawa, 2013) and provides an explanation for why living things do not become extinct even when mutation rates exceed the threshold. The theory and model predict that the error frequency is significantly higher in the lagging DNA strand, since a more complex system is used in the synthesis of the lagging strand compared to that of the leading strand (Furusawa and Doi, 1992). Indeed, biased mutagenesis was observable in the lagging strand in *Escherichia coli (E. coli*) when both strands are synthesized by polα (Iwaki et al., 1996).

**Figure 1:**
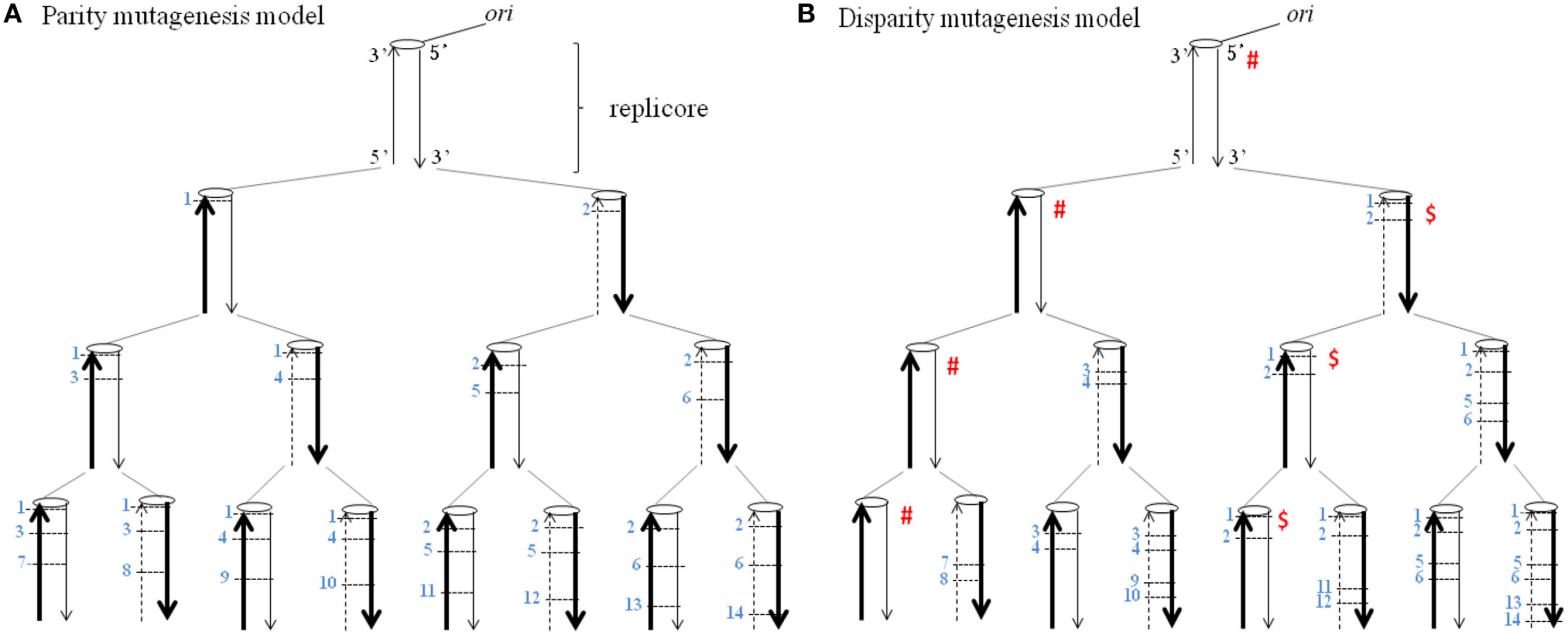
**The distribution of mutations according to the deterministic model of a single replicore is shown**. A broad arrow indicates a template DNA strand, a thin arrow indicates a newly synthesized leading strand and a dashed thin arrow indicates a newly synthesized lagging strand. Each number on the side of a short horizontal bar indicates a base substitution at a different site. The ori indicates the replication origin. **(A)** Parity mutagenesis model. One mutation per a single replication is evenly introduced into both daughter DNAs. **(B)** Disparity mutagenesis model. Two mutations are introduced exclusively in the lagging strand per a single replication. Notice that for instance, in the family line of the genomes with the symbol marked # or $, the genotype is guaranteed forever.

Figure 1 shows the basic principle of the disparity-mutagenesis model of evolution. The replicore has one replication origin (*ori*) at the upper end of a linear DNA. When it replicates semiconservatively, random point mutations (SNPs) are deterministically introduced exclusively to the lagging strand and each mutation once introduced is certainly inherited. Conclusions obtained from the pedigree shown in the disparity-mutagenesis model (Figure 1B) are as follows. (1) Each replication produces two daughter DNAs. One has the same genotype as its parent, while the other inevitably has a different genotype by newly added two mutations in each replication. (2) The ancestral genotype with zero mutations is guaranteed forever. (3) Any genotype that appeared in the past is inherited and its existence is guaranteed at any down-stream generation.

In other words, the creation of diversity with a guarantee of principal can be realized. Within the model, mutations are postulated to be neutral or mildly deleterious, and all mutants are able to survive. In nature, however, there exist neutral, mildly deleterious and deleterious mutations. When an unchanged environment continues for a long period, the ancestral genotype with zero mutations can be used. When the environment changes, an adequate mutant is selected from previously provided mutants. The newly selected mutants can continue to produce their offspring in the same manner. It is noticeable that the ancestral genotype is always guaranteed independently of mutation rates. However, when environments change again dramatically, the ancestral individual is no longer able to adapt to the new environment. Increasing mutation rates would be beneficial to adapt to new environments. When mutation rates are too high, however, there is little chance to find appropriate individuals from preexisting mutants because they have accumulated an excess of deleterious mutations which might cause a significant decrease of fitness. Generally speaking, however, one can predict that the threshold must be significantly increased with increasing mutation rates in the disparity mutagenesis model.

In fact, our previous studies clearly showed that Eigen's error-threshold (Eigen et al., 1989) in his quasi-species model moved up or disappeared when a mixture of two kinds of error-less and error-prone polymerases was used (Aoki and Furusawa, 2003; Furusawa, 2012). Our experimental conditions used in this study correspond to realizing the fidelity difference between the leading and lagging strands in DNA replication.

## Acceleration of evolution using digital organisms with disparity mutagenesis

Using a “hill-climbing” game, we showed that the intracellular coexistence of error-prone mutator DNA polymerase and normal high fidelity DNA polymerase increased the mutation threshold and accelerated evolution (Aoki and Furusawa, 2001).

Genetic algorithms can be used as a metaphor of evolution of living things. We constructed a DNA-type genetic algorithm, the “neo-Darwinian algorithm” (Wada et al., 1993). Genetic algorithms can be used as a metaphor of evolution of living things. Knapsack optimization problems mimic the evolutionary process. There are a given kinds of objects with weights and values determined at random. The objective is to maximize the total value of objects placed in a knapsack, subject to a loading-weight limitation, and taking a plural number of the same object is not allowed (Goldberg, 1989). At appropriate mutation rates and disparity mutagenesis, the algorithm clearly resolved a knapsack optimization problem, and the threshold was considerably increased compared to the conventional parity mutagenesis model in which mutations occur evenly in the lagging and leading strands (cf. Figure 1A). For instance, when the total mutation rate was 2.32%, the population became extinct under parity mutagenesis conditions. In contrast, when using the disparity mutagenesis model, the population quickly adapted and solved even when the total mutation rate was 8% (Wada et al., 1993). Knapsack problems were resolved more effectively when the fidelity differences between both strands were larger, meaning that the algorithms evolved well (Wada et al., 1993).

## Acceleration of evolution using disparity-mutators of living microorganisms

As *E. coli* used in our earlier experiments had no F-factor, sexual conjugation was not involved. *E. coli* has a circular DNA genome consisting of 4.6 × 10^6^ bp and about 4300 genes. Both DNA strands are synthesized by polα and proofreading is done by dnaQ. Mutant *dnaQ49* is a temperature-sensitive mutator. At 37°C, the proofreading activity is deleted. Therefore, *dnaQ49* appears to reflect the net errors in the base-paring process to which polα is committed without the help of dnaQ. Our previous experiment with *dnaQ49* showed that the error frequency in the lagging strand synthesis was about 100 times higher than that of the leading strand (Iwaki et al., 1996). *dnaQ49* mutators were cultured at 37°C to introduce mutations and followed by selection with gradually increasing concentration of different antibiotics at 24°C, where no mutator phenotype was expressed. Surprisingly, they were able to make colonies in the presence of saturated concentrations of all antibiotics tested. Their phenotypes thus acquired were stable and maintained for a long period (Tanabe et al., 1999). The super-ampicillin-tolerant *dnaQ49* strain (resistant to 30 mg/ml; the highest concentration testable) thus obtained was highly sensitive to other antibiotics comparable to intact *E. coli* or intact *dnaQ49*. It can be concluded that the *dnaQ49* adapted exclusively to ampicillin given as a selection pressure. This is consistent with other studies which describe bacterial disparity mutators with high adaptabilities to different environments and genome-wide base changes (Itakura et al., 2008; Loh et al., 2010).

Haploid yeast, *Saccharomyces cerevisiae*, has sixteen chromosomes, 1.2 × 10^7^ bp genomic DNA and about 6000 genes. It replicates by budding asexually. Hundreds of *ori*s exist in the total genome. *S. cerevisiae* pol3 (polδ) is specialized to synthesize the lagging strand and *Pol3-01* is a mutator with deleted proofreading activity. Thus, biased mutagenesis may occur in the lagging strand, which is one of the disparity-mutators. *Pol3-01* was cultured with gradually increasing temperature in order to isolate temperature-resistant strains. Two temperature-resistant strains that produce colonies at 40°C were isolated. Genetic analysis showed that at least two stepwise mutations were necessary for acquiring this phenotype. Namely, the first mutation at the hot1 locus provided 38.5°C-resistant properties, and by the addition of the second mutation (not identified) the mutant was able to grow at 40°C (Shimoda et al., 2006).

There are several other experiments in which yeast disparity-mutators displayed high adaptabilities to different conditions (Abe et al., 2009a,b,c; Park et al., 2011; Kato and Park, 2012). Beyond unicellular organisms, disparity-mutators in mice also can repeat generations with increased incidence of carcinogenesis, while the evidence of apparent adaptive evolution has not yet been reported (Albertson et al., 2009; Uchimura et al., 2009).

Collectively, the following conclusions can be deduced from experiments using disparity-mutators of living organisms: (1) Growth rates of disparity mutators and of the intact cells are nearly equal, when cultured in normal conditions. This feature would be an essential condition for evolution experiments, because a delayed cell-cycle will work against the rate of accumulating mutations, resulting in delayed adaptive evolution; (2) The principle of the disparity mutagenesis model may be applicable to all living prokaryotes and eukaryotes; (3) A prolonged period of high mutation rates, during which most likely average mutation rates exceed the threshold value, does not necessarily lead to the death of organisms; (4) To attain a final intended phenotype, a number of appropriate mutations should be introduced in a correct order (Furusawa, 2013) and most probably, genome-wide changes with mutations are necessary. These might be the reasons why final phenotypes were so stable.

## Discussion

### Implications of selection, sexuality and the disparity mutagenesis for biological evolution

Due to the molecular mechanism of DNA replication, which produces disparity in mutagenesis, higher organisms might maintain population survival and still evolve irrespective of high mutation rates. In contrast, population genetics postulates that truncation selection will play a central role, yet it cannot fully resolve the problem.

With that perspective, the role of sex was pointed out by Crow (2000). In fact, our simulations with the neo-Darwinian algorithm showed that adequate degrees of cross-over and sex brought about increased fitness, the delay of extinction time or sometimes the avoidance of extinction (Wada et al., 1993). It has been shown that Paramecia can become extinct by the accumulation of harmful mutations when cultured for a long period without conjugations (Holmes and Holmes, 1986). As mentioned above, however, our disparity mutagenesis model predicts an increased mutation rate threshold; i.e., in the case of the knapsack problem without sex (Wada et al., 1993), in our evolution experiments with living microorganisms (Tanabe et al., 1999; Shimoda et al., 2006) and in the case of human tumor expansion (Furusawa, 2013). These systems were carried out without relation to sex. Accordingly, from the above-mentioned viewpoint of population genetics, these results must be explained mainly by the effect of truncation selection which would be expected to be difficult.

Recently, it has been reported that spontaneous deleterious mutations per generation per diploid in humans are estimated as 2.2 (Keightley, 2012). If this estimation is correct, then L is 0.89 and high genetic load should have led to human extinction. Recent studies of human population genetics have tried to explain this discrepancy by focusing on natural selections other than truncation selection. Most notably, relative selection and stabilizing selection seem to be attractive, if appropriate parameters are selected. In nature, however, various kinds of selection pressure might act on thousands of mutated sites scattered throughout the genome. Moreover, we have to pay attention to the interaction among sites. Overall then, it can be said that much more work will be necessary to clarify this problem.

Here, we would like to stress the different positions between population genetics and our disparity mutagenesis model as we approach the mechanism of evolution. Population genetics deals with a relatively large population and very large time scale, such as 4 billion yeas of the history of life, or 6 million years of the history of humans. From this view, evolution is considered to progress extremely slowly. By contrast, our disparity mutagenesis model deals with the non-equilibrium phase of evolution. We deal with numerable number of generations and focus on the mechanism for how species can avoid extinction when faced with rapid environmental change. A possible strategy to avert crisis would be increase mutation rates in order to reproduce many variants within a short period. As a function of increased mutation rates, excess deleterious mutations might accumulate in the population, indicating that the risk of extinction must increase. We believe the clues to resolve this contradiction lie in the molecular mechanism of DNA replication.

The primary role of the disparity mutagenesis model on evolution would be not to provide a broad repertoire of candidates for future evolution, but to positively guarantee the genetic information existing at the present generation and to transfer it intact to the next generation by means of the leading strand with high fidelity.

Let us suppose that there are 10,000 replicores in a human diploid cell and the number of mutations introduced into each replicore per generation is 0.01. As this value is far less than the threshold value, the effect of disparity mutagenesis cannot be expected in a single replication unit. At a cellular level, however, mutations per generation run up to 100. This number is well in accordance with the actually observed values in case of humans, which are too high to keep the average fitness and the population size. Mutations are thought to be intensively introduced at the late stage of spermatogenesis (Crow, 2000). The point worth noting for the present discussion is not the timing of mutagenesis, but the total number of mutations that has been introduced during the maturation of germ cells in both parents.

According to the disparity mutagenesis model, when the mutagenesis occurs exclusively in the lagging strand in the parent germ cells, the preexisting genotypes are guaranteed by the leading strand. This means that when the genomic DNA replicates, all of the preexisting genotypes in genome DNA are kept intact in terms of replicore. This situation would not be changed even when the mutation rate increases up to 10-fold or more, such as might occur at in a hot spot. However, the lagging strand will contribute to providing new genotypes. Of course, any new genotype once produced by the lagging strand will be guaranteed in descendants by virtue of the leading strand (Figure 1B). Shuffling of genomic DNA by sex and chromosomal recombination would produce chromosomes consisting of various combinations of mutated replicores. These replicores with enlarged diversity are drifting in a population. By these means, the disparity mutagenesis model predicts increased possibility for producing better zygotes than the conventional random-mutation model, even when mutation rates are very high.

The triadic synergy effect of disparity mutagenesis, sex and natural selection would make it possible to increase the chance to make a quality zygote that harbors fewer deleterious mutations or even has increased fitness. Different kinds of selections will indirectly contribute to decreasing average mutation rates in the population through removing effectively deleterious mutations from the population. Characteristics of the disparity-mutagenesis model are the guarantee of parental genetic information and the increase of error-threshold, both of which reflect a “robustness” of our model. This mechanism is thought to function as a basic principle independently of natural selection. The methodology of population genetics after taking the concept of disparity mutagenesis into consideration would serve as a key toward resolution of this difficult problem.

### Role of the lagging-strand-biased mutators

As expected, the parity-mutator in yeasts, in which the proofreading activities of both DNA polymerase (polδ and, polε) are deleted, could not make colonies (Morrison and Sugino, 1994). The 3′ → 5′ exonucleases of both DNA polymerase δ and ε participate in correcting errors of DNA replication in *S. cerevisiae*. This destructive effect of parity-mutagenesis on a living cell was also deduced from the experiments with the neo-Darwinian algorithm, in that the parity-mutator with high mutation rates definitively resulted in rapid decrease of fitness scores or even the extinction of the population (Wada et al., 1993). The loss of growth in the parity mutator yeasts likely comes about because as excess mutations are evenly introduced in both strands, the established quality genotype for surviving would be easily canceled by additional mutations.

For a variety of reasons, we have never used a disparity mutator which carries out biased-mutagenesis in the leading strand. Eight evolutionary experiments using mutator strains of eukaryotes have been reported. They exclusively used mutators with lagging-strand-biased mutagenesis (Shimoda et al., 2006; Abe et al., 2009a,b,c; Uchimura et al., 2009; Park et al., 2011; Kato and Park, 2012). Each of their experimental protocols was based on the essential idea of our disparity theory of evolution, the lagging-strand-biased mutagenesis (Furusawa and Doi, 1992, 1998). There are two examples in which the lagging-strand-biased mutagenesis has been observed in natural environment. The first one is an experiment conducted in natural mutator with the purpose of occurrence of the lagging-strand biased mutagenesis using *dnaQ49* mutator of *E. coli* (Iwaki et al., 1996).

The other one is a molecular evolution study, which suggests a frequent decrease of proofreading function of pol delta in the lagging strands during the mammalian evolutionary process. It has been shown that the speed of mammalian molecular clocks is faster than those of other vertebrates. It was presumed that the cause of their faster molecular clock might be due to the frequent replacements of key amino acids in the proofreading domain of polδ, indicating that the proofreading activities of polδ might have gone up and down repeatedly in the past (Katoh et al., 2005). Accordingly, in these species including humans, the speed of evolution might change frequently during the evolutionary process. An observation supporting this idea is that amino acid substitution rates in mammals are higher in the proof-reading domains of polδ compared to those of other vertebrates. It is also known that in all vertebrates examined, amino acid substitution rates of the polymerase domain of polδ and polε, and those of the proofreading domain of polε are low, compared to those of the proofreading domain of mammalian polδ (Katoh et al., 2005; Furusawa, 2012). Similar observations were obtained in the case of birds (K. Katoh, personal communication).

In vertebrates, although the precise role of polδ and polε in replicating DNA has not yet been clarified, it is presumed that ancestors of mammals and birds might have many occurrences of the disparity mutator phenotype, where excess mutations might be introduced exclusively in the lagging strand and each time evolution might be accelerated (Furusawa, 2012). Consequently, it can be predicted that the fidelity of the leading strand of existing vertebrates including humans might remain high.

In fact, there was a research on a mutator yeast with the leading-strand-biased mutagenesis done by the present author and his colleagues. Although the mutant grew normally (Shimoda et al., 2006), this evolutionary experiment was not proceeded due to the fact that this mutator phenotype for DNA replication goes against the basic concept of our model, the lagging-strand-biased mutagenesis (Figure 1B). However theoretically said, the “leading-strand mutator” may also show a high adaptability.

## Concluding remarks

Genomic DNA not only codes genetic information but also works as an excellent genetic algorithm which can resolve dynamic and complicated optimization problems in changing environments. Its driving force for resolving the problems (pursuing evolution) comes from the fidelity difference between the lagging and leading strands. The key enzyme which creates and controls the fidelity difference would be the proofreading domain of polδ. In plain words, “the ultimate cause of the precise heredity would be traced in the leading strand of high fidelity, and that of evolution in the lagging strand of low fidelity” (Furusawa, 2012).

To the extent that the disparity mutagenesis model works, humans will not become extinct. More likely, humans might well adapt to drastic environmental changes by increasing the mutation rates in the lagging strand, and might be able to adjust the speed of evolution depending on the situations. This may well be true for other species as well.

### Conflict of interest statement

The author declares that the research was conducted in the absence of any commercial or financial relationships that could be construed as a potential conflict of interest.
